# Glutamate Receptors and Glioblastoma Multiforme: An Old “Route” for New Perspectives

**DOI:** 10.3390/ijms20071796

**Published:** 2019-04-11

**Authors:** Lorenzo Corsi, Andrea Mescola, Andrea Alessandrini

**Affiliations:** 1 Department of Life Sciences, University of Modena and Reggio Emilia, Via G. Campi 287, 41125 Modena, Italy; 2 CNR-Nanoscience Institute-S3, Via Campi 213/A, 41125 Modena, Italy; andrea.mescola@nano.cnr.it (A.M.); andrea.alessandrini@unimore.it (A.A.); 3 Department of Physics, Informatics e Mathematics, Via G. Campi 213/a, 41125 Modena, Italy

**Keywords:** glioblastoma multiforme, glutamate receptors, mechanobiology, cell migration, α-amino-3-hydroxy-5-methyl-4-isoxazole propionic acid (AMPA), *N*-methyl-d-aspartate (NMDA), focal adhesion complex (FAK), protein kinase B (Akt)

## Abstract

Glioblastoma multiforme (GBM) is the most aggressive malignant tumor of the central nervous system, with poor survival in both treated and untreated patients. Recent studies began to explain the molecular pathway, comprising the dynamic structural and mechanical changes involved in GBM. In this context, some studies showed that the human glioblastoma cells release high levels of glutamate, which regulates the proliferation and survival of neuronal progenitor cells. Considering that cancer cells possess properties in common with neural progenitor cells, it is likely that the functions of glutamate receptors may affect the growth of cancer cells and, therefore, open the road to new and more targeted therapies.

## 1. Glioblastoma

Gliomas are the most frequent type of primary tumors of the central nervous system in adults. Among them, glioblastoma multiforme (GBM) is the most malignant brain tumor in adults, with complex biology and poor prognosis. Typically, primary GBMs, the most frequent type of glioblastomas, develop de novo without pre-cancerous lesions, whereas the secondary GBMs progress from low-grade or anaplastic astrocytomas. From the histological point of view, primary and secondary glioblastomas are quite similar, but they differ from a genetic point of view. Indeed, secondary GBMs carry the *IDH1* mutations associated with hypermethylation of the phenotype which are absent in primary GBMs. Moreover, primary GBMs generally show *EGF* gene amplification, together with the phosphatase/tensin homolog (PTEN) mutation.

Unfortunately, although many improvements were achieved in cancer biology, for almost all patients with GBM, life expectancy remains within 18 months [1,2]. Up to now, the combination of radiotherapy with the oral alkylating agent temozolomide (TMZ) represents the first major advance in GBM therapy, increasing the mean lifetime survival by 1.8 years [3]. Indeed, the addition of the anti-vascular endothelial growth factor A antibody, bevacizumab, to standard treatment with TMZ improves only the progression-free survival but does not affect the overall survival [4]. Notwithstanding this, the drug was found to be active in patients who did not respond to other standard treatments [5]. A common hallmark of all cancer cells is their ability to migrate and to metastasize other tissues. Although extracranial metastases are extremely rare, GBM can migrate into the healthy brain tissue, leading to local or distant cancer dissemination, rendering surgical resection of the tumor impossible and conferring to these cells the ability to elude the efficacy of irradiation and chemotherapy [6,7]. Therefore, understanding the molecular mechanisms underlying its aggressive behavior and cell migration is fundamental in order to develop novel therapeutic strategies to treat GBM patients with better outcomes.

It was shown that the alteration of glutamate secretion and signaling in GBM and glioma might play a pivotal role in the invasion and tumor growth [8,9]. In addition, GBM secretes a considerable amount of glutamate in the microenvironment nearby the cancer, causing excitotoxicity in normal neurons [10] and increasing expressions of glutamate transporters and glutamine synthetase. This latter effect might be responsible for the recruitment of tumor-associated microglia/macrophage (TAM), which mediates tumor cell survival through secretion of IL-10 and Vascular-Endothelial Growth Factor (VEGF) [11]. Therefore, the glutamate receptor system could be an important target in GBM therapy.

### 1.1. Oncogenic Pathways in GBM

GBM is a disease where genetic, epigenetic, and post-translational aspects cooperate together in a complex and well-orchestrated series of events leading to the oncogenic transformations of neuronal cells. It is commonly accepted that GBMs origin can be ascribed to the so-called stem-cell theory, which is split into two main hypotheses: the astrocyte dedifferentiation theory and the glioblastoma stem-cell (GSC) theory. Both are characterized by the presence in astrocytic glioma of a subpopulation of cells which exhibit neuronal stem-cell-like properties such as multipotentiality, and the ability to self-renew or to form neurospheres in vitro. Moreover, experiments on a GBM mouse model showed that neural stem cells and oligodendrocyte precursor cells also have the potential to form glioblastomas, although with distinct molecular characteristics, reinforcing the stem-cell theory. The important role played by cytosolic free Ca^2+^ must also be underlined. Indeed, Ca^2+^ is involved in a number of signaling events related to its ability to maintain cell proliferation in tumor cell lines and to commit embryonic stem cells toward a neuronal phenotype [12]. The most frequently altered pathway in GBM and cancer in general involves both tyrosine kinase receptors (RTKs) and serine/threonine kinases [13] such as the epidermal growth factor receptor (EGFR). These receptors bind different growth factors (GFs), and, upon activation, they dimerize and change their conformation. This event activates the kinase function of the RTK resulting in downstream signaling cascades. EGFR signaling is involved in several biological functions such as proliferation, differentiation, survival, and migration of all types of central nervous system cells [14]. In GBM, the oncogenic properties of EGFR are characterized by an uncontrolled increase in phosphorylation activity, resulting in uncontrolled cell proliferation. EGF also regulates the serine/threonine phosphorylation pathways such as the phosphatidylinositol- 4,5-bisphosphate 3-kinase/phosphatase and tensin homolog/serine–threonine kinase Akt (PI3K/PTEN/Akt) which is implicated in the pathogenesis of GBM [15,16]. In addition, EGFRs and receptor tyrosine kinases (RTKs) regulate the activities of Ras, an overexpressed oncogene in GBM [17]. Indeed, the Ras/Raf pathway is modified in GBM. Ras is a guanosine binding protein that becomes active when bound to Guanosine Triphosphate (GTP). Upon activation, Ras/Raf phosphorylates a series of kinases including the mitogen-activated protein kinase MAPK family, which in turn regulates downstream target genes and cell activities. Alterations of the Ras/MAPK pathway are responsible for abnormal cell growth and proliferation, as well as cell invasion [18]. In addition, it was demonstrated that the upregulation of Ras/Raf/MAPK and PI3k/Akt/mTOR pathways is a key element promoting TMZ resistance [19,20], which in turn enables a selection of slow-cycling glioma stem cells (GMCs) able to escape chemotherapy’s effect [21], originating a chemo-resistant cell subpopulation.

### 1.2. Glioma Cell Motility and Invasion

The ability of GBM cells to infiltrate the healthy tissue nearby is a crucial point both in disseminating cancer cells and as a “strategy” to escape from irradiation and chemotherapy. GBM cell infiltration is a complex and intricated mechanism which is accomplished basically via two main routes, blood vessels and axons [22]. However, due to such complexity, its biology is still poorly understood; however, at the same time, it is of great importance for developing novel therapeutic approaches to treat the disease. The composition of the extracellular matrix (ECM) significantly influences the ability of glioma cells to migrate. We can assume that the stiffness gradient is one of the major driving forces of cell motility; therefore, the GBM migrates toward a more rigid micro-environment according to a durotaxis phenomenon [23]. In addition, it was shown that matrix crosslink-enhanced ECM tension was able to promote tumor cell migration progression [24,25]. Integrins are catalytic heterodimeric transmembrane glycoproteins responsible for cell–ECM interactions. Once activated, integrins and the formation of downstream focal adhesion complexes (FAK) enhance the migration [26] and transduce mechanical signals into biochemical signals. Among other integrins, β1 seems to play an important role in cell migration and tumor spreading. In fact, it was shown that β1 integrin protein levels were strongly and specifically downregulated in cells cultured on soft matrix, causing a decrease in fibrotic deposition and, possibly, in tumor malignancy [27]. FAKs and integrin β1 are active and overexpressed in gliomas, and their expression correlates with the tumor grade [28,29]. Interestingly, the finding that the α-amino-3-hydroxy-5-methyl-4-isoxazole propionic acid (AMPA) subunit GluR1 associates with integrin β1 suggests that α-amino-3-hydroxy-5-methyl-4-isoxazole propionic acid receptors (AMPARs) might act as membrane-associated cytoskeleton anchors for localized signaling at focal adhesion complexes [30] and, therefore, might play an important role in GBM cell migration. It should be underlined also that the cancer microenvironment plays an important role in GBM cell migration. Indeed, several factors, such as hypoxia [31], deregulated EFGR pathway, and increased intracellular Ca^2+^ concentration, are able to activate the migratory machinery in glioma cells [32]. Both ionotropic and metabotropic glutamate receptors interact with and/or regulate the proteins involved in such activities, reinforcing the idea that glutamate receptors could be also a key protein target for new anti-metastatic drugs.

## 2. Glutamate Receptors and Cancer

In the last decade, considerable evidence describing the involvement of glutamate in tumor development was reported in both neural and non-neural cancer tissues of both malignant and benign origin. Both metabotropic [33] and ionotropic [34] glutamate might act as a growth factor and as a signal mediator in both autocrine and paracrine fashions [35]. The glutamate receptor system is the main excitatory network of the central nervous system (CNS). It is composed of three sub-families, two ligand-gated ion channels (ionotropic receptors), the *N*-methyl-d-aspartate (NMDA) receptors and the α-amino-3-hydroxy-5-methyl-4-isoxazole propionic acid (AMPA)/kainate receptors, and one metabotropic receptor (mGluR) [36]. The receptor subunit composition of both NMDA and AMPA ion channels confer specific pharmacological properties to these receptors. Both receptors mediate the entrance of Na^+^ and Ca^2+^ but with different activation kinetics. Indeed, NMDA activity is linked to the synaptic plasticity involved in learning and memory [37]. The rapid desensitization kinetics of AMPA receptors mediates the fast excitatory synaptic transmission, which is fundamental in shaping and driving synaptic plasticity [38]. The rise of intracellular Ca^2+^ concentration is associated to a number of Ca^2+^-dependent signal transduction pathways involved in cell proliferation such as Akt, [39], ERK/MAP kinase [40], and PKA [41] pathways. Interestingly, GBMs possess the ability to release glutamate in the nearby environment, which in turn interacts with the specific Ca^2+^-permeable AMPA receptor, allowing Ca^2+^ entrance into the cells and promoting cell migration and proliferation through Akt activation. The release of glutamate by a transient increase of the intracellular Ca^2+^ concentration is also associated with the presence of high levels of ATP in the extracellular matrix, contributing in part to the influx of external Ca^2+^ through the ionotropic ATP-gated receptor P2X7 [42]. In addition, it was shown that the cystine/glutamate antiporter xc(-), which exchanges extracellular cystine for intracellular glutamate, is upregulated in GBMs [43]. Thus, in the GBM environment, the simultaneous presence of high levels of ATP and the upregulation of the xc(-) transporter and the expression of Ca^2+^-permeable AMPA receptors generate a detrimental scenario that allows GBM to grow and migrate into brain tissue.

The increased level of glutamate in the brain also has clinical relevance. Indeed, patients with GBM showed a concentration of glutamate ranging from 100 to 600 μM, responsible for seizure and excitotoxicity in cells close to the tumor site [44]. Moreover, it is well known that the microenvironment is crucial for the regulation of several processes involved in tumor formation and progression, such as the epithelial–mesenchymal transition, and the maintenance of GSCs by enhancing their self-renewal capacity. The role of microenvironment also becomes important in the regulation of pH, which, in GBMs, resulted to be around 6.1, whereas, in normal brain, is around 7.1. This is mainly due to the high metabolic rate of tumor tissue. This acidic condition contributes to the GBM tumor growth and the self-renewal of GSC [45]. The metabotropic glutamate receptor (mGluR) is a G-protein-coupled receptor localized in both pre- and post-synaptic regions in CNS. However, mGluRs are not exclusive to the CNS; they are ubiquitously expressed in different non-neuronal human tissues [46], such as the skin [47], liver, heart [48], and adrenal gland [49]. Metabotropic glutamate receptors are composed of eight subtypes, mGluR1–8, subdivided in turn into three groups (group I, II, and III) based on sequence homology and intracellular G-protein coupling activity [50]. Once activated, they modulate signal transduction through the stimulation of second messengers such as the phospholipase C/inositol triphosphate/diacylglycerol pathway or by inhibiting the adenylate cyclase pathway [41,51]. The mGluRs, in the CNS, control the postsynaptic neuronal response to glutamate, modulating both NMDA and AMPA receptor activity, as well as cellular proliferation, growth, migration, survival, and calcium-mediated cellular homeostasis [52]. In particular, group I, characterized by mGluR1 and mGluR5, is coupled to Gαq, which causes stimulation of Phospholipase C beta (PLCβ) and activation of protein kinase C (PKC), resulting in phosphorylation of downstream targets. Group II, consisting of mGluR2 and mGluR3, and group III, composed of mGluR4 and mGluR6–8, are coupled to Gαi/o and, therefore, responsible for the inhibition of adenylyl cyclase, which potentiates or attenuates different pathways including the mitogen-activated protein kinase (MAPK) and phosphoinositide 3-kinase (PI3K)/Akt signaling pathways [37,53].

### 2.1. Metabotropic Glutamate Receptor and GBM

**Figure 1:**
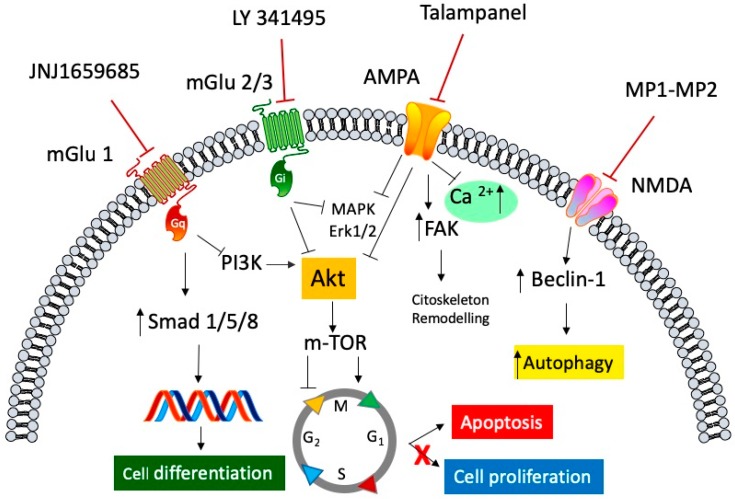
General representation of the signaling pathways involved in the antagonism of both ionotropic and metabotropic glutamate receptors in anti-cancer mediated effects. Selective metabotropic glutamate receptor 1 (mGluR1) antagonist JNJ1659685 elicited its anti-cancer activity, by decreasing cell proliferation, through the inhibition of the phosphoinositide 3-kinase/protein kinase B (PI3K/Akt) pathway, and by increasing cell differentiation through the activation of the transcription factors small mother against decapentaplegic transcription factor (Smad) 1/5/8. LY 341495, an mGluR 2/3 selective antagonist, blocks the activation of both the mitogen-activated protein kinase (MAPK)/extracellular signal-regulated kinase (ERK)1/2 cascade and the Akt/mammalian target of rapamycin protein (mTOR) pathway, resulting in arrest of the cell cycle and an increase in apoptosis. Talampanel, a selective negative allosteric α-amino-3-hydroxy-5-methyl-4-isoxazole propionic acid (AMPA) modulator, inhibits the rise of intracellular calcium concentration, and inhibits both ERK1/2 and Akt phosphorylation, slowing down the cell proliferation, and increasing cellular stiffness through the activation of focal adhesion kinase (FAK) protein. The *N*-methyl-d-aspartate (NMDA) antagonists, MP1 and MP2, two memantine derivative compounds, exert their antiproliferative activity by modulating Beclin-1 binding protein, which increases the autophagic processes of glioblastoma cell line.

As already mentioned, GBM releases a high amount of glutamate extra-synaptically, thus acting as a trophic factor, increasing the proliferation and migration of glioma cells. Studies on messenger RNA (mRNA) expression and protein level amount of mGluR in the GBM cell line and primary culture from GBM patient resections revealed that group II was the most expressed [54]. Moreover, the finding by Ciceroni et al. [55] that the low levels of mGluR3 mRNA expression in tumor specimens correlated with a better survival rate in GBM patients reinforced the idea that the use of an antagonist of mGluR3 might be an important strategy to pursue [54]. In this context, the study of the mGluR2/3 antagonist LY 341495 on the U87MG glioma cell line showed a dramatic inhibition of cell proliferation, through a negative regulation of gap 1/synthesis (G1/S) phase transition, without affecting (or involving) apoptosis [56]. Interestingly, D’Onofrio at al. confirmed these data also on primary cultures from human GBM; however, in this case, cell growth was restored after washing out LY 341495, indicating that the antiproliferative effect was cytostatic, rather than cytotoxic [57]. This effect was due to different molecular mechanisms, involving the ERK1/2 phosphorylation pathway on differentiated GBM cells and an increase in Smad 1/5/8 phosphorylation, which is a downstream effector of the morphogenetic protein (BMP), which in turn is relevant in glial cell differentiation of glioblastoma stem cells (GSC) [58] (Figure 1). Moreover, Yelskaya et al. (2013) suggested a possible synergism between mGluR2/3 and EGFR, through a common activation of Akt and MAPK pathways, since the combination of mGluR2/3 antagonist LY 341495 and Iressa, an EGFR inhibitor, is more efficient to inhibit proliferation and migration and to induce apoptosis in U87MG cells [59]. Recently, Dalley at al. [60] found a selective mRNA overexpression of mGluR1 in GBM cell lines, suggesting that mGluR1, when overexpressed, might act as a proto-oncogene by promoting dysregulated proliferation and survival of glioma cells. To reinforce this idea, the authors evaluated the effect of the noncompetitive selective mGluR1 antagonist JNJ16259685. The selective antagonist was able to convert Hs683 cells grown in soft agar from an anchorage-independent phenotype to an anchorage-dependent phenotype, indicating that Hs683 cells treated with this antagonist exhibited reduced metastatic and tumorigenic characteristics in vivo [60]. A molecular explanation for the antitumor effects elicited by antagonists acting on mGluR1 could reside in the reduction of phosphorylation of PI3K and mTOR target p70 S6K. Indeed, in the U87MG cell line, the inhibition of mGluR1 activity resulted in the inhibition of Phosphoinositide-3-kinase (PI3K) and Akt/protein kinase B (PKB) phosphorylation, together with a decreased expression level of phosphorylated (p)-mTOR and P70 S6K, without affecting the expression of PTEN [61]. As described in the previous section, upregulation of EGFR stimulates survival signaling through the Ras/Raf/MAPK and PI3K/Akt/mTOR pathways and is involved in GMC TMZ resistance [20,21] (Figure 1). In this context, Gli-1 protein, a downstream target of Hedgehog signaling (Hh), might play an important role. In fact, Gli-1 is involved in several cellular functions such as cell proliferation [62], survival, and migration [63], as well as cancer cell stemness and self-renewal [64], and its overexpression correlates with malignancy of several cancers including GBM [65]. Interestingly, Zhang et al. showed that a selective agonist of mGluR3 VU0155041 suppressed cell proliferation of the glioblastoma cell line LN through the suppression of Gli-1 transcription factor, suggesting this pathway as a possible key element in the mGluR4 control of GBM cell growth [66], suggesting this receptor subtype as a potential target for glioblastoma therapy.

### 2.2. Glutamate Ion Channels in GBM and Cell Migration

Tumor cell migration is a complex event characterized by highly dynamic and coordinated biological processes, comprising cell adhesion and detachment, cell motility, and cell invasion. Adhesion and motility are directly related via the interactions of extracellular matrix (ECM) with integrins, FAK, and the actomyosin system. Invasion, instead, requires the secretion, by tumor cells, of proteolytic enzymes such as matrix metalloproteinases (MMPs), able to degrade the surrounding extracellular matrix. Ionotropic glutamate receptors are cation-specific ion channels which allow the influx of monovalent ions and also divalent ions such as Ca^2+^. In GBM, Ca^2+^ signaling is really important, since it is involved in a number of functions ranging from the motility of cancer stem cells to the motility and growth of GBM cells [12]. AMPA receptors are tetrameric ion channel receptors which can be composed of four different subunits named GluR1, GluR2, GluR3, and GluR4. In the presence of GluR2, the receptor complex becomes impermeable to Ca^2+^, and this configuration is the most widespread in the healthy brain [67]. In GBM, GluR2 is poorly represented, whereas the AMPA receptor configuration permeable to Ca^2+^, formed only by GluR1 and GluR4 subunits, is overexpressed [8,68]. As mentioned above, the overexpression of GluR1 and its correlation with the high expression of integrin β1 indicate an important role of GluR1 in cell migration and adhesion in GBM. In addition, Piao et al. showed a direct correlation between the expression of AMPARs and the level of glioma cell migration, both in vitro and in vivo. This finding is supported also by the evidence that a high level of GluR1 is associated with high levels of FAKs, and that glutamate increased Rac1/GTPase activity, an intracellular protein involved in the assembly of integrin adhesion complex [30] (Figure 1). Moreover, as represented in Figure 1, the important role played by MAPK and Akt signaling in GBM cell proliferation was proposed to be a downstream event of AMPA receptor activation through an increase in intracellular Ca^2+^ [34]. This assumption is supported also by the finding that GluR2 overexpression inhibits glioblastoma cell proliferation by inactivating MAPK and inducing apoptosis of tumor cells [69]. Further evidence comes from the fact that GluR1 knock-down inhibits AMPAR-mediated activation of the MAPK pathway and decreases glioma cells proliferation [70]. As already discussed, it is well known that the tumor microenvironment plays a crucial role in the survival and propagation of cancer cells [71]. Both Ca^2+^ and EGFRs are key elements in this environment, upregulating the plasma membrane antiporter xc(-), which are essential for the exchange of extracellular cysteine and intracellular glutamate [32] and enhancing the hypoxia-inducible factor (HIF) activity, respectively [72], thus promoting, partially through AMPAR, tumor cell migration. EGFRs, whose expression is deregulated in GBM, were shown to interact also with NMDA receptors, and in particular with the NR2B subunit [73]. NMDA receptors are heterotetrameric ion channels composed of different subunits: GluN1, a glycine/d-serine binding subunit, and GluN2 (GluN2A-D) or GluN3 (GluN3A-B), a glutamate-binding subunit [74]. The phosphorylation of NR2B by EGFR increases the intracellular Ca^2+^ concentration and, therefore, tumor growth and spreading. Recently, Wan-Soo et al. reported the effect of memantine, a GluN1 antagonist, on a glioblastoma cell line. Interestingly, the drug, which is used in Alzheimer’s and Parkinson’s diseases, was able to reduce cell proliferation and increase autophagy in the T-98G cell line. These effects were mediated by the interaction with GluN1, by increasing the expression of Beclin-1, which in turn regulates the autophagic pathway. [75]. Moreover, novel memantine-derived drugs, called MP1 and MP2, were found to inhibit cell proliferation and increase apoptosis in the U87MG glioblastoma cell line, without affecting significantly the cell viability of primary human whole blood cells [76].

### 2.3. Glutamate Receptor and Biomechanic Features of GBM

As mentioned in previous sections, it was demonstrated that tumor microenvironment affects cancer cells properties including proliferation, death, and motility [71]. Several studies in the last decades revealed how living cell behavior can also be affected by mechanical and other biophysical cues from the cell microenvironment [77,78] giving rise to a dynamic and reciprocal exchange of mechanical and biochemical information. These mechanical signals play a key role in a global comprehension of disease processes as they may contribute to the pathogenesis and/or be used as a target for therapeutics.

**Figure 2:**
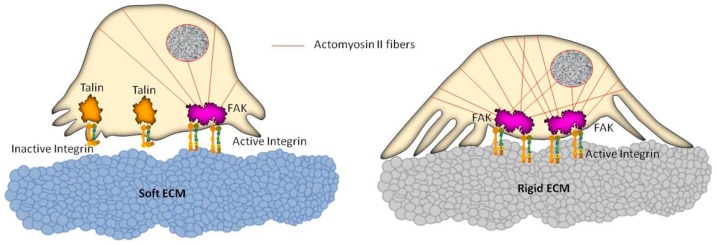
Schematic representation of cell reorganization on soft and rigid extracellular matrix (ECM) substrates. The actomyosin II system connected to focal adhesion complexes generates intracellular contractile forces which are transmitted to the underlying substrate, following a dynamic and mutual information exchange that can lead to changes in cell motility. A soft substrate (left) entails reduced activation of integrins, not allowing the formation of stress fibers within the cell. Conversely (right), a rigid substrate promotes the integrin activation, as well as a remarkable recruitment of actin filaments, leading to a global reinforcement of the cell, thus affecting the cell migration ability.

Focusing specifically on GBM, it was demonstrated that invasive properties of tumor cells can be regulated by ECM stiffness, which provides an active and dynamic microenvironmental cue able to mediate the contractile actomyosin bundles [79]. In particular, it was proposed that GBM cells may induce a stiffening of microenvironmental properties during growth and spreading and that these remodeling phenomena can give rise to a reciprocal mechanical signaling exchange with those cells that promote tumor invasion (Figure 2). Investigations about the role of ECM-based substrates with different stiffness on GBM behavior led Ulrich et al. [24] to assert, for the first time, a remarkable stiffness-dependent behavior in glioma cell migration and proliferation. These findings assume an outstanding relevance when considering the different mechanical microenvironmental features associated with healthy and tumor brain tissue. In fact, it is generally recognized that GBM tumors and their surrounding stroma are stiffer if compared with healthy brain tissue. Interestingly, they also demonstrated the feasibility to bypass their dependence on stiffness by pharmacologically inhibiting the actin motor protein non-muscle myosin II (NMMII). These interesting results highlight the key role of both mechanical signals present in the tumor microenvironment and the molecular complexes which are able to sense and process these signals, opening new perspectives for understanding and manipulating glioma cell physiology, as well as for therapeutics.

Umesh et al. went further, revealing that the microenvironmental rigidity dependency of GBM tumor cell behavior occurs via the alteration of EGFR-dependent signaling [80]; in particular, it was demonstrated that an enhanced environmental stiffness modulates expression and phosphorylation of EGFR and its downstream effector Akt, also affecting a wide range of other signals along the EGFR pathway involved in proliferation. Moreover, they found that rigid substrates not only affect GBM cell proliferation but also promote the passage through the G1/S checkpoint of the cell cycle, consistent with an EGFR-dependent process [81]. They finally revealed how the pharmacological inhibition of EGFR, Akt, or PI3 kinase pathways results in a significantly less stiffness-dependent proliferation, confirming that mechanical cues such as ECM substrate stiffening promotes GBM proliferation by acting on EGFR signaling pathways.

As highlighted in previous sections, even if the overall mechanism remains not completely clear, it is understood that an over-activation of glutamate receptors is associated with neurodegenerative disease processes. In particular, it was found that there is a strong correlation between the NMDA receptor degeneration and the dynamic remodeling of a wide range of intracellular Rho GTPases, including Rac, Rho, and Cdc42, and other actin-binding proteins [82]. In this context, studies about mechanical changes of neuroblastoma cells connected with glutamate-mediated neurodegeneration were recently performed [83]. In 2013, Zou et al. evaluated, using atomic force microscopy (AFM)-based measurements, a huge increase in the Young’s modulus of cortical neurons exposed to NMDA, which was correlated to high hydrostatic pressure inside the neurons [84]. More recently, Fang and coauthors focused their attention on dynamic actin filament reorganization of neuroblastoma cells as a consequence of NMDA exposure; they showed a direct link between NMDA treatment and cell mechanical property changes, including an enhanced level of surface roughness and higher stiffness [83]. These findings, coupled with the mechanical approach, allow dynamic monitoring of changes occurring at the single-cell level, providing not only information about the neurodegenerative mechanism at the basis of several pathological processes but also an innovative diagnostic tool useful for drug screening and to develop new therapeutic agents.

## 3. Future Perspective

All these important findings strongly highlight the possibility to candidate both ionotropic and metabotropic glutamate receptors as pharmacological targets in GBM treatment. However, up to now, the only drugs that reached the phase I or II of clinical trials are the selective AMPA antagonist talampanel and the NMDA antagonist memantine. In particular, talampanel was found to increase the median survival of patients with GBM (20.3 months versus 14.6 months) in a cohort study of 60 patients [85]. However, the clinical use of talampanel in GBM did not reach the final phase of clinical trials and is not in the guidelines for the treatment of GBM, either alone or in association with other therapies. Memantine was proposed for the treatment of GBM in phase I clinical trial (NCT01260467), in a repurposing drug strategy for recurring GBM. Unfortunately, due to the poor number of patients enrolled or to the poor patient accrual, there are no available results. However, another phase I clinical trial is still ongoing, aimed at evaluating the anticancer activity of memantine associated with TMZ (NCT01430351). As described previously, the acidic microenvironment plays an important role in both GSC maintenance and GBM growth. Recently, the ability of new series of allosteric NMDAR inhibitors was reported, which showed greater potency in acidic conditions [86]. Although these inhibitors were designed primarily for traumatic brain injuries or stroke, the acidic feature of the GBM microenvironment could be exploited to target new drugs, acting on glutamate receptors, selective only for GBM. Despite the encouraging results obtained involving the glutamatergic system in glioma and GBM biology, the number of clinical trials on these types of cancer is really low. The reasons for the lack of clinical trials, in addition to the difficulty of transferring the preclinical test to the clinical one, such as the controlled environment of cultured cells, could be ascribed to two main points. The first one might reside in the possible unwanted side effects elicited by specific antagonists on this system, such as loss of memory, mood alteration, ataxia, and hallucination, in particular when using ligands acting on ionotropic receptors. The second one, regarding the metabotropic receptors, relies mainly on the needs of further research, aimed at better understanding the activity and the molecular mechanism of specific positive or negative allosteric ligands acting on mGluRs, in parallel to the investigation of their safety and tolerability profile. However, it must be underlined that, from a pharmacological point of view, the evaluation of the degree of risk and benefit and the therapeutic window should be carefully taken into account, especially if we consider the poor prognosis and lethality of GBM.

Finally, the evidence that the positive allosteric modulation of metabotropic glutamate receptors mGlu1 and mGlu4 was shown to be neuroprotective, whereas the negative allosteric modulation, acting on mGlu3, was able to interfere with cell proliferation, differentiation, and chemoresistance [87] strongly suggests the mGluRs as promising targets, and reinforces the idea of a possible use, in the future, of new specific ligands acting on the glutamatergic receptor system, alone or in combination with other drugs, in the treatments of GBM. Moreover, the ability of NMDA receptors to modulate the mechanical properties of the tumor cells highlights the possibility to have more tools to study the dynamic features of tumor cells within their microenvironments and, therefore, to better understand the cancer biology both for a diagnostic purpose and to develop new anticancer strategies and/or agents.
